# A multiplex TaqMan real-time PCR assays for the rapid detection of mobile colistin resistance (*mcr-1* to *mcr-10*) genes

**DOI:** 10.3389/fmicb.2024.1279186

**Published:** 2024-03-13

**Authors:** Xinran Gong, Guang Yang, Wei Liu, Di Wu, Chunyuan Duan, Xinjing Jia, Zhiqiang Li, Xiaocang Zou, Renfeng Yu, Dayang Zou, Yong Wang

**Affiliations:** ^1^School of Public Health, China Medical University, Shenyang, China; ^2^Chinese PLA Center for Disease Control and Prevention, Beijing, China; ^3^The 5th Medical Center of General Hospital of Chinese People’s Liberation Army, Beijing, China; ^4^National Institute for Communicable Disease Control and Prevention, Beijing, China; ^5^Centre for Evidence-based Chinese Medicine, Beijing University of Chinese Medicine, Beijing, China

**Keywords:** colistin, the *mcr* genes, multiplex TaqMan real-time PCR, rapid detection, multidrug-resistant

## Abstract

**Objective:**

Recently, 10 plasmid-mediated mobile colistin resistance genes, *mcr-1* to *mcr-10*, and their variants have been identified, posing a new threat to the treatment of clinical infections caused by Gram-negative bacteria. Our objective was to develop a rapid, sensitive, and accurate molecular assay for detecting *mcr* genes in clinical isolates.

**Methods:**

The primers and corresponding TaqMan-MGB probes were designed based on the sequence characteristics of all reported MCR family genes, multiplex Taqman-MGB probe-based qPCR assays were developed and optimized, and the sensitivity, specificity and reproducibility of the method were evaluated. The assay contained 8 sets of primers and probes in 4 reaction tubes, each containing 2 sets of primers and probes.

**Results:**

The standard curves for both the single and multiplex systems showed good linearity (R^2^ > 0.99) between the starting template amount and the Ct value, with a lower limit of detection of 10^2^ copies/μL. The specificity test showed positive amplification results only for strains containing the *mcr* genes, whereas the other strains were negative. The results of intra-and inter-group repeatability experiments demonstrated the stability and reliability of the newly developed method. It was used to detect *mcr* genes in 467 clinically-obtained Gram-negative isolates, which were multidrug-resistant. Twelve strains containing the *mcr* genes were detected (seven isolates carrying *mcr-1*, four isolates carrying *mcr-10*, and one isolate carrying *mcr-9*). The products amplified by the full-length PCR primer were identified by sequencing, and the results were consistent with those of the multiplex qPCR method.

**Conclusion:**

The assay developed in this study has the advantages of high specificity, sensitivity, and reproducibility. It can be used to specifically detect drug-resistant clinical isolates carrying the *mcr* genes (*mcr-1 to mcr-10*), thus providing a better basis for clinical drug treatment and drug resistance research.

## 1 Introduction

The growing problem of bacterial drug resistance poses a serious threat to public health, especially with the emergence of multidrug-resistant (MDR) organisms posing challenges for the treatment of nosocomial infections (Lancet, 2022). Polymyxin, a peptide antibiotic, was withdrawn from clinical use in the 1980s because of its side effects which included nephrotoxicity and neurotoxicity. The emergence of MDR organisms and the lack of new antibiotics have led to the reintroduction of polymyxins as the “last resort “for treating infections caused by MDR gram-negative bacteria (Trimble et al., 2016; Soman et al., 2021).

In November 2015, the first plasmid-mediated colistin resistance gene, *mcr-1*, was found in *Escherichia coli* isolated from a pig farm in China (Liu et al., 2016). Studies have shown that the *mcr-1* gene could be detected in patients, animals, food, and the environment (Liu et al., 2023; Shahzad et al., 2023). Plasmids carrying the *mcr-1* gene have conjugation and transfer abilities, contributing to the stability and persistence of colistin resistance (Liu et al., 2016). Subsequently, the *mcr-1* gene and its variants have been reported in many countries, with 113 variants identified in 10 families of the *mcr* gene. The coexistence of the *mcr* genes and other drug-resistance genes increases the likelihood of the emergence of pan-drug-resistant superbugs (Karim et al., 2023; Zhang et al., 2023). The prevalence of multidrug resistance can lead to an increased rate of hospital-acquired infections with limited treatment options while increasing the length of hospital stays, mortality, and costs (Strich and Palmore, 2017; Manandhar et al., 2022).

To further regulate the rational use of antibiotics and prevent the emergence and widespread occurrence of drug resistance, it is essential to rapidly detect bacteria carrying the *mcr* genes and provide a basis for monitoring and clinical drug treatment. The TaqMan minor groove binder (MGB) probe fluorescence technique, is a quantitative real-time polymerase chain reaction (qPCR) approach that is currently the most rapid and reproducible assay for the quantitative and qualitative detection of nucleic acid molecules; it provides faster results than conventional PCR methods and often without the use of high-risk reagents (Kutyavin et al., 2000). The TaqMan MGB qPCR method is widely used in areas such as transgenic and gene expression studies, and for the detection of infectious and genetic diseases (Wang et al., 2021; Jin et al., 2022). To effectively detect isolates carrying *mcr* genes, we compared all MCR family genes in the database, designed seven sets of primers and TaqMan-MGB probes based on the sequence comparison results, and introduced the primer and probe sets of 16S rRNA as an internal control for amplification (Center for Disease Control and Prevention, 2011). A multiplex probe-based qPCR assay was developed and optimized to detect all MCR family genes in four reaction tubes, which was evaluated on a sample set of 467 multidrug-resistant clinical isolates.

## 2 Materials and methods

### 2.1 Bacterial strains

Eight clinical multidrug-resistant isolates with whole gene sequencing were selected for the specific experiment, and information on the resistance genes of the isolates was obtained with good representativeness (see Table 1 for detailed information on the isolates and drug resistance). In addition, 467 clinically multidrug-resistant isolates, collected previously, were used for the overall evaluation of the assay.

**Table 1:** Information on clinical multidrug-resistant isolates for specific experiments.

No.	Species	Source	Drug-resistant genes
1	*Pseudomonas aeruginosa* N12122	Clinical isolate	*bla*_OXA-50_, *bla*_VIM-2_, *bla*_PAO_, *aph(3′)-IIb*, *aac*A4*, aph(6)-Id, str*A*, aac(6′)-Ib-cr, qnr*VC1*, fos*A
2	*Klebsiella pneumoniae* N12106	Clinical isolate	*bla*_SHV-2_, *aac(6’)Ib-cr, aac(3)-IId, aad*A16*, qnr*B6*, fos*A*, arr-3, sul*1*, tet*(D)
3	*Acinetobacter baumannii* N12118	Clinical isolate	*bla*_OXA-23_, *bla*_OXA-66_, *Amp*C-β-lactamase, *bla*_TEM-1B_, *arm*A*, aph(6)-Id, aph(3″)-Ib, mph*(E)*, tet*(B)*, sul*1
4	*Serratia marcescens* N12145	Clinical isolate	*bla*_KPC-2_, *bla*_CTX-M-14_, *bla*_SRT-1_, *bla*_CTX-M-3_, *aac(3)-IId, aac(6′)-Ic*
5	*Escherichia coli* N12139	Clinical isolate	*aac(3)-IId, aph(3″)-Ib, mdf*(A)*, mph*(A)*, sul*1*, sul*2*, tet*(A)*, dfr*A17
6	*Enterobacter cloacae* N12169	Clinical isolate	*bla*_TEM-1B_, *oqxA, oqxB, mdf*(A)*, fos*A*, bla*_IMP-8_*, aac(6′)-Ib-cr, bla*_CTX-M-3_
7	*Proteus mirabilis* N12160	Clinical isolate	*bla*_TEM-1_, *aad*A2*, aad*A5*, aac(3)-IIb, aph(3′)-Ia, aph(3″)-IIb, aph(6)-Id, sul*1*, sul*2*, dfr*A17*, tet*(J)*, cat*I
8	*Stenotrophomonas maltophilia* N12146	Clinical isolate	Sm*qnr, bla*_L1_, *sul*1*, dfr*A12*, aad*A2*, aac(6′)-Ib-cr*

### 2.2 Design and synthesis of primers and probes

All available MCR family genes were downloaded from the Reference Gene Catalog of the National Center for Biotechnology Information (NCBI), including *mcr-1* to *mcr-10* genes and their variants. Partial sequences were compared again using the CLC sequence viewer 8 (Qiagen Aarhus, Denmark) based on the published phylogenetic tree results of MCR family genes constructed according to the maximum likelihood ratio (Ling et al., 2020). Conserved regions with no mutation points were selected, and standard primers and Taqman-MGB probes were designed using Primer Express 3.0.1 software according to the principles of multi-PCR primer design (Hawkins and Guest, 2017). Primers were designed for maximum coverage of *mcr* gene variants and using degenerate bases if necessary. The primer set of 16S rRNA was used as an internal control for amplification. Primer-Blast was used to evaluate the specificity of the primers and AutoDimer software was used to assess primer-dimer production between primer groups (Vallone and Butler, 2004). Multiple PCR was developed by mixing primer groups according to the evaluation results. All primers were synthesized by Beijing Tsingke Biotech Co., Ltd. (Beijing, China).

### 2.3 Optimization of the reaction system of multiplex real-time PCR

Before mixing the primers in the multiplex PCR reaction system, each primer group (including the probe) was individually optimized for maximal amplification efficiency. Using the recombinant plasmid as the template, a 25 μL reaction system was developed, and the primer concentration (100–500 nmol/L), TaqMan probe concentration (50–500 nmol/L), and annealing temperature (56.6–62.6°C) were optimized individually. Three replicates were used for each experiment. The optimal concentrations of primers and probes were selected according to the Ct values and fluorescence signal intensity of the amplification curves to ensure that the amplification efficiency of all targets was between 90 and 110%, with an R^2^ value of ≥0.985. Finally, a multiplex PCR reaction optimization system was developed, and that showed similar Ct values and amplification efficiencies compared to those for single-duplex PCR. The instrument used for the experiments was a CFX 96 Connect Real-Time PCR Detection System (Bio-Rad, United States), and the reagents used were Premix Ex Taq™ (Probe qPCR) purchased from Takara Biomedical Technology Co., Ltd. (Beijing, China).

### 2.4 Standard curves

After synthesizing the amplified target sequence, it was cloned into the pUC57 recombinant plasmid and identified by sequencing as a positive standard. The concentration of the recombinant plasmid was determined and converted into copy numbers according to the following formula:


$$\text{Copy number}=\frac{\left(6.02\times 10^{23}\right)\times \left(\frac{ng}{\mu l}\times 10^{-9}\right)}{\left(\text{DNA}\;\text{length}\times 660\right)}$$

The plasmids were 10-fold serially diluted from 10^9^ copies/μL to 10^2^ copies/μL. A 25 μL reaction system was set up according to the optimized reaction conditions with three replicate wells. The amplification efficiencies of the single and multiplex systems were also evaluated, and standard curves were generated.

### 2.5 Specificity

Eight clinical multidrug-resistant bacteria without the *mcr* genes were used for the experiments (see Table 1 for detailed information on the isolates and drug resistance). *Escherichia coli* containing the recombinant plasmid was used as a positive control. Nucleic acids were extracted using the Qiagen DNA Mini Kit (Qiagen, Hilden, Germany), following the manufacturer’s instructions. The original nucleic acid solution was diluted 100-fold for amplification, and deionized water was used as no template control (NTC). All samples were analyzed using the multiplex fluorescence qPCR method.

### 2.6 Sensitivity

The plasmids were 10-fold serially diluted from 10^3^ copies/μL to 10^1^ copies/μL. A 25 μL reaction system was developed according to the optimized reaction conditions with three replicate wells, and negative controls. Means and standard deviations (SD) were calculated. The minimum copy concentration at which a Ct value occurs is usually considered the limit of detection.

### 2.7 Reproducibility

To evaluate the stability of the assay, five different concentrations of standards were prepared ranging from 10^7^ copies/μL to 10^3^ copies/μL. For intra-group repeatability tests, three wells were repeated for each dilution; for inter-group repeatability tests, three reactions were repeated 1 week apart. Standard deviation and coefficient of variation (CV) were calculated to analyze intra-and inter-group differences.

### 2.8 Clinical sample testing

Nucleic acids from 467 clinical multidrug-resistant isolates were extracted using the Qiagen DNA Mini Kit (Qiagen, Hilden, Germany) following the manufacturer’s instructions. The extracted nucleic acids were diluted 100-fold and screened for *mcr-1* to *mcr-10* genes using the newly developed multiplex fluorescence qPCR method. For positive results, PCR amplification was performed using standard full-length primers, and the products were identified by sequencing. The results of both methods were analyzed to evaluate the usefulness of the new method.

## 3 Results

### 3.1 Specific primers and probes

**Figure 1:**
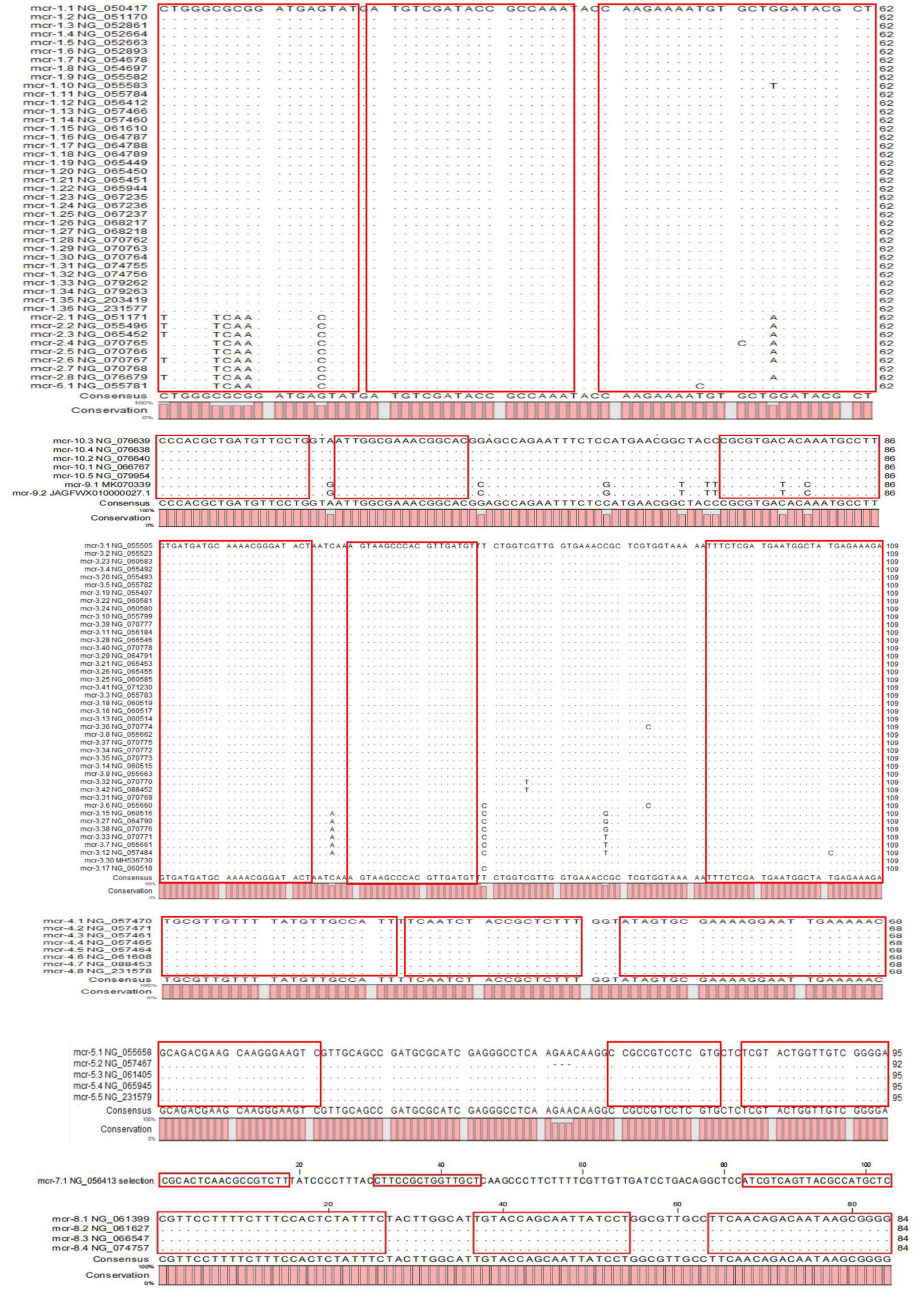
The results of sequence alignment.

The results of the phylogenetic tree of MCR family genes showed high levels of similarity between the *mcr-1*, *mcr-2*, and *mcr-6* genes and between the *mcr-9* and *mcr-10* genes. Therefore, we designed primers and probes targeting the amplification of *mcr-1/2/6*, *mcr-3*, *mcr-4*, *mcr-5*, *mcr-7*, *mcr-8*, and *mcr-9/10*, and introduced the primer set of 16S rRNA as the internal control for amplification. The results of sequence alignment are shown in Figure 1. Based on results of the AutoDimer Check software, the eight sets of primers and probes were divided into four tubes, each contained two sets of primers and probes. Sequence information and the grouping of the primers and probes are shown in Table 2. It is worth noting that there are two forward primers for amplifying the *mcr-1/2/6* gene, and some primers and probes contain mixed bases.

**Table 2:** Primers and probes were designed to detect plasmid-mediated colistin resistance genes (*mcr-1* to *mcr-10*).

Tube	Target	Primers / Probes name	Sequence (5′-3′)	Length (bp)	Tm (°C)	Final concentration (nmol/L)	Reference
A	*mcr-1* *mcr-2* *mcr-6*	*mcr-1/2/6-F1*	CTGGGCGCGGATGAGTAT	62	58.8	200	This study
		*mcr-1/2/6-F2*	CTGGGTCAAGATGACTAT		49.7	200	
		*mcr-1/2/6-R*	AGCGTATCH*AGCACATTTTCTTG		58.8	200	
		*mcr-1/2/6-P*	FAM-ATGTCGATACCGCCAAA-MGB		68	100	
	16S rRNA	16S rRNA-F	TGGAGCATGTGGTTTAATTCGA	159	59.1	200	Control CfD Prevention
		16S rRNA-R	TGCGGGACTTAACCCAACA		58.6	200	
		16S rRNA-P	HEX-CACGAGCTGACGACAR*CCATGCA-BHQ		72	200	
B	*mcr-3*	*mcr-3-F*	GTGATGATGCAAAACGGGATACT	109	58.9	200	This study
		*mcr-3-R*	TCTTTCTCATAGCCATTCATCGAG		58.5	200	
		*mcr-3-P*	HEX-AGTAAGCCCACGTTGATGT-MGB		70	200	
	*mcr-9* *mcr-10*	*mcr-9/10-F*	CCCACGCTGATGTTCCTG	86	57.1	200	This study
		*mcr-9/10-R*	AAGGCATTK*GTR*TCACGCG		55.5	200	
		*mcr-9/10-P*	FAM-ATTGGCGAAACGGCAC-MGB		69	100	
C	*mcr-4*	*mcr-4-F*	TGCGTTGTTTTATGTTGCCATT	68	59	200	This study
		*mcr-4-R*	GTTTTTCAATTCCTTTTCGCACTAT		58	200	
		*mcr-4-P*	FAM-TCAATCTACCGCTCTTT-MGB		68	100	
	*mcr-7*	*mcr-7-F*	CGCACTCAACGCCGTCTT	103	59.6	200	This study
		*mcr-7-R*	GAGCATGGCGTAACTGACGAT		58.8	200	
		*mcr-7-P*	HEX-CTTCCGCTGGTTGCT-MGB		69	200	
D	*mcr-5*	*mcr-5-F*	GCAGACGAAGCAAGGGAAGTC	95	60	200	This study
		*mcr-5-R*	TCCCCGACAACCAGTACGA		58.5	200	
		*mcr-5-P*	FAM-CCGCCGTCCTCGTG-MGB		69	100	
	*mcr-8*	*mcr-8-F*	CGTTCCTTTTCTTTCCACTCTATTTC	84	58.8	200	This study
		*mcr-8-R*	CCCCGCTTATTGTCTGTTGAA		58.7	200	
		*mcr-8-P*	HEX-TGTACCAGCAATTATCCT-MGB		69	200	

^*^R = A or G; K = G, T; H = A, C, T.

### 3.2 Optimization of the reaction system of multiplex qPCR

After optimization tests, optimal reaction conditions were obtained for the multiplex PCR system (25 μL): 12.5 μL Premix Ex Taq™ (Probe qPCR), 2 μL mixed standard primers (10 μmol/L), 0.25 μL FAM-labelled probe (10 μmol/L), 0.5 μL HEX-labelled probe (10 μmol/ L), 2 μL DNA template and the remaining volume was made up with deionized water. The optimized amplification program was 95°C for 30s, followed by 40 cycles of 95°C for 5 s and 57.8°C for 30s, with the fluorescent signal collected at 57.8°C. The groups were prepared by placing primer sets amplifying *mcr-1/2/6* and 16S rRNA, *mcr-3* and *mcr-9/10*, *mcr-4* and *mcr-7*, and *mcr-5* and *mcr-8* in the same reaction tube; the concentrations of the primers are detailed in Table 2. Positive (1 × 10^5^ copies/μL plasmid standard) and no-template (water) controls were included in each plate.

### 3.3 Standard curve for single and multiplex qPCR

**Figure 2:**
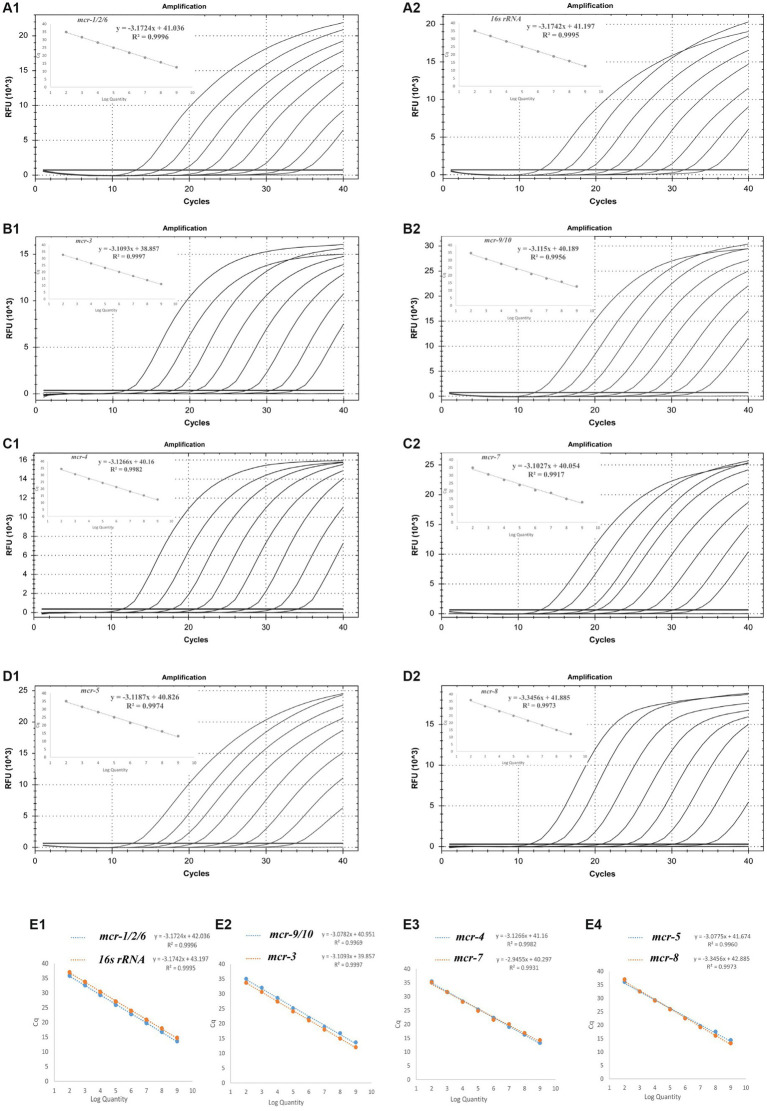
Standard curve and the amplification efficiencies of the single (A1–D2) and multiplex systems (E1–E4).

The amplification efficiency of primers and probes in single and multiple systems was evaluated, and the amplification and standard curves were plotted. Good linearity was observed between the starting template concentration and the Ct values for the eight sets of primers and probes in both the single and multiplex systems, with the correlation coefficient R^2^ ranging from 0.9960–0.9997 and amplification efficiencies between 90 and 110%. The amplification efficiency of the multiplex qPCR assay is similar to that of a single qPCR assay, which meets the requirements. A standard curve is shown in Figure 2.

### 3.4 Specificity of the multiplex qPCR assay

**Figure 3:**
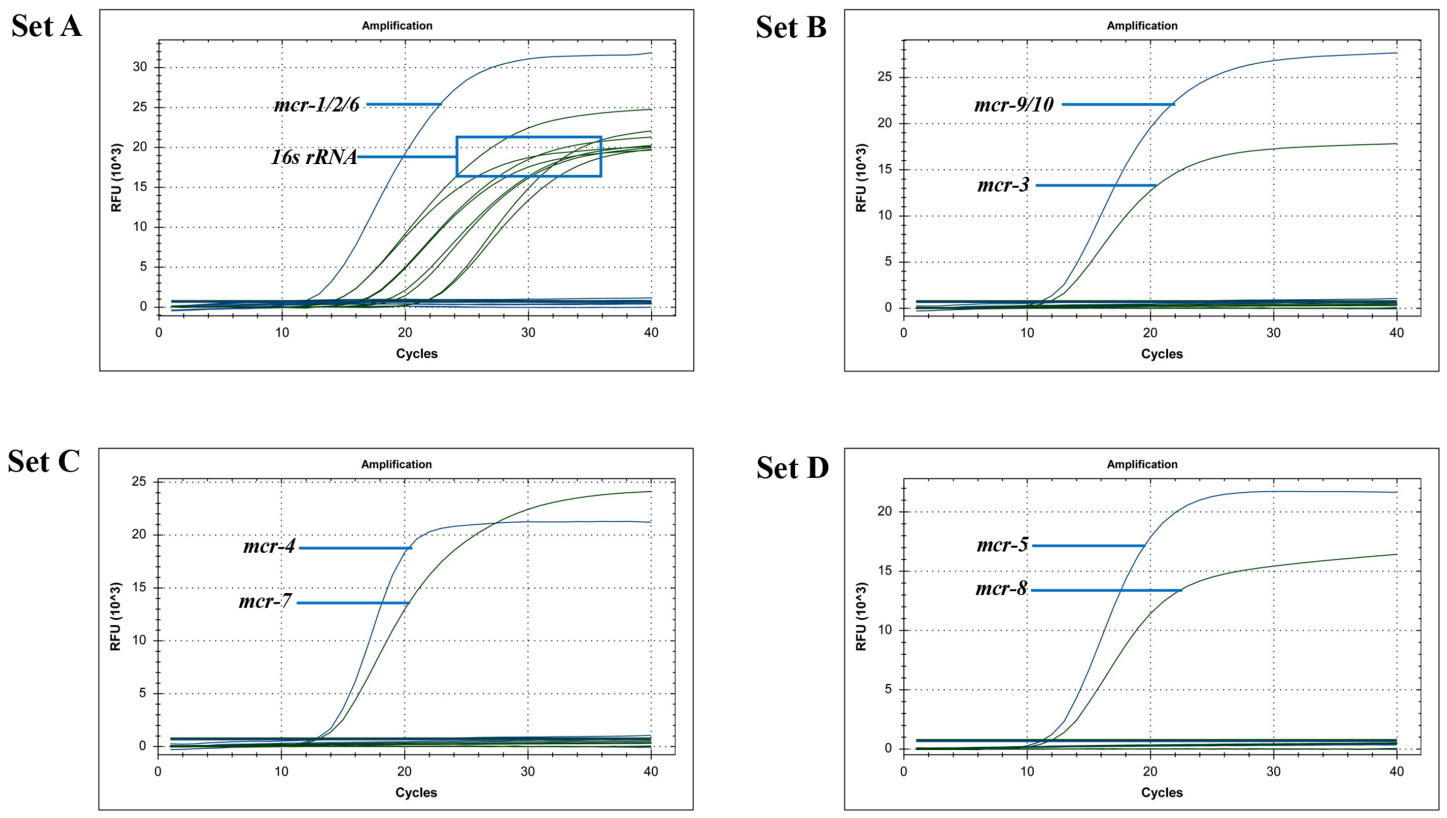
Specificity of the multiplex qPCR assay.

After uploading the designed primers to the NCBI database for specific comparison, we confirmed that the primers can only amplify genes related to *mcr*. The genomic DNA of *Escherichia coli* containing the recombinant plasmid of the *mcr* gene and eight clinical multidrug-resistant isolates (see Table 1) were used as templates to verify the specificity of the developed multiplex qPCR assay. The results showed that only nucleic acid samples from *Escherichia coli* containing recombinant plasmids were positive, while nucleic acid samples from other clinical isolates and negative controls were negative, indicating good specificity of the assay. The amplification results are presented in Figure 3.

### 3.5 Sensitivity of the multiplex qPCR assay

Three concentration gradients of recombinant plasmid standards, from 1 × 10^3^ copies/μL to 1 × 10^1^ copies/μL, were used as templates to verify the sensitivity of the multiplex qPCR assay. The results showed that when the template concentration was 10^2^ copies/μL, all primers and probes showed amplification curves in the three parallel controls, and when the template concentration was 10^1^ copies/μL, only some of the primers and probes showed amplification curves in the three parallel controls. From the perspective of assay integrity, the minimum detectable limit of multiplex qPCR is 10^2^ copies/μL.

### 3.6 Reproducibility of the multiplex qPCR

Reproducibility tests were performed using five concentration gradients of recombinant plasmid standards as templates. The results showed CVs ranging from 0.12 to 1.34% for intra-group reproducibility tests and from 0.10 to 1.91% for inter-group reproducibility, indicating that the developed fluorescent qPCR method is reproducible. The detailed values are listed in Table 3.

**Table 3:** Reproducibility assay of TaqMan real-time PCR.

Plasmid (copies)	*mcr-1/2/6*						*mcr-9/10*					
	Intra-			Inter-			Intra-			Inter-		
	Mean	SD	CV	Mean	SD	CV	Mean	SD	CV	Mean	SD	CV
10^7^	19.12	0.02	0.12%	19.05	0.07	0.38%	18.39	0.14	0.79%	18.08	0.26	1.46%
10^6^	22.09	0.04	0.16%	22.09	0.08	0.35%	21.18	0.04	0.19%	21.00	0.16	0.76%
10^5^	25.07	0.06	0.23%	25.19	0.09	0.36%	24.23	0.04	0.16%	24.12	0.10	0.42%
10^4^	28.32	0.06	0.20%	28.47	0.11	0.37%	27.41	0.09	0.34%	27.47	0.14	0.50%
10^3^	31.84	0.16	0.51%	31.82	0.03	0.10%	30.82	0.29	0.95%	30.79	0.04	0.14%

Plasmid (copies)	*mcr-3*						*mcr-4*					
	Intra-			Inter-			Intra-			Inter-		
	Mean	SD	CV	Mean	SD	CV	Mean	SD	CV	Mean	SD	CV
10^7^	18.34	0.07	0.36%	18.47	0.15	0.83%	19.37	0.06	0.31%	19.29	0.09	0.49%
10^6^	21.48	0.06	0.26%	21.70	0.32	1.46%	21.10	0.20	0.94%	21.05	0.31	1.46%
10^5^	24.58	0.15	0.59%	24.83	0.32	1.30%	24.49	0.07	0.29%	24.44	0.10	0.40%
10^4^	27.99	0.20	0.71%	28.12	0.24	0.85%	27.38	0.18	0.64%	27.44	0.24	0.86%
10^3^	31.20	0.38	1.22%	31.36	0.38	1.22%	31.06	0.07	0.23%	30.93	0.26	0.84%

Plasmid (copies)	*mcr-5*						*mcr-7*					
	Intra-			Inter-			Intra-			Inter-		
	Mean	SD	CV	Mean	SD	CV	Mean	SD	CV	Mean	SD	CV
10^7^	19.29	0.06	0.29%	18.87	0.36	1.91%	18.9967	0.18824	0.99%	19.05	0.06	0.31%
10^6^	21.80	0.05	0.21%	21.60	0.18	0.83%	21.1633	0.09074	0.43%	20.83	0.29	1.41%
10^5^	24.88	0.11	0.44%	24.85	0.16	0.63%	23.8133	0.31943	1.34%	23.81	0.08	0.35%
10^4^	28.13	0.04	0.13%	28.16	0.08	0.28%	27.1333	0.07234	0.27%	27.12	0.03	0.11%
10^3^	31.21	0.14	0.43%	31.34	0.17	0.54%	30.64	0.07	0.23%	30.60	0.10	0.32%

Plasmid (copies)	*mcr-8*						16S rRNA					
	Intra-			Inter-			Intra-			Inter-		
	Mean	SD	CV	Mean	SD	CV	Mean	SD	CV	Mean	SD	CV
10^7^	18.33	0.13	0.71%	18.24	0.12	0.64%	18.9967	0.18824	0.99%	19.05	0.06	0.31%
10^6^	21.43	0.08	0.35%	21.45	0.15	0.70%	21.1633	0.09074	0.43%	20.83	0.29	1.41%
10^5^	24.81	0.07	0.28%	24.75	0.20	0.79%	23.8133	0.31943	1.34%	23.81	0.08	0.35%
10^4^	28.22	0.13	0.46%	28.12	0.11	0.37%	27.1333	0.07234	0.27%	27.12	0.03	0.11%
10^3^	31.24	0.14	0.46%	31.30	0.24	0.76%	30.64	0.07	0.23%	30.60	0.10	0.32%

### 3.7 Identify the specific type of *mcr* gene

This detection system is sufficient to meet the need for rapid screening of *mcr* genes in samples/strains. For further identification of different *mcr* gene types, full-length gene amplification can be performed on samples/strains containing relevant *mcr* genes, and the sequencing results of amplified products can be uploaded to the NCBI database for comparison to determine specific *mcr* gene types.

### 3.8 Clinical isolate detection

We used a newly developed multiplex qPCR method to screen for the presence of the *mcr* gene in 467 multidrug-resistant clinical isolates. This method detected 7 isolates carrying the *mcr-1.1* gene (6 in *Escherichia coli* and 1 in *Klebsiella pneumoniae*), 1 isolate carrying the *mcr-9.1* gene (in *Enterobacter cloacae*), and 4 isolates carrying the *mcr-10.1* gene (2 in *Enterobacter ludwigii* and 2 in *Enterobacter asburiae*) in clinical strains. The sequencing results of the PCR products were confirmed to be *mcr*-related genes by Sanger sequencing (electrophoresis results in the Supplementary Figure S1), which were consistent with those of the multiplex qPCR assay. This suggests that the method can be used to screen for *mcr* genes in clinical isolates.

## 4 Discussion

Enterobacteriaceae exhibit polymyxin resistance through the acquisition of plasmid-mediated MCR family genes. Many scholars at home and abroad have developed methods for the detection of *mcr* genes including standard PCR (Liu et al., 2016), multiplex PCR for *mcr-1* to *mcr-9* genes (Borowiak et al., 2020), SYBR Green fluorescent qPCR (Mentasti et al., 2021), TaqMan fluorescent qPCR (Irrgang et al., 2016) and other methods. The ring-mediated isothermal amplification (LAMP) assays for rapid detection of *mcr-1* to *mcr-5* genes in colistin-resistant bacteria are available. The LAMP method is highly sensitive and specific compared with the standard PCR method. However, due to the diversity of *mcr* genes, a single LAMP cannot detect all potential target genes, which provides incomplete information for nucleic acid detection. For samples containing more than one *mcr* gene, which has been reported in several cases, the sensitivity and specificity of the multiplex LAMP assay are relatively poor (Zhong et al., 2019). This situation has been reported many times so far and cannot be ignored (Zhang et al., 2018; Lu et al., 2020). One study introduced a Quad-PCR method for rapid and reliable detection of the common *mcr-1*, *mcr-3*, *mcr-8*, and *mcr-10* genes in clinical samples (Hu et al., 2021a). A multi-PCR assay for the detection of mobile colistin resistance genes (*mcr-1*, *mcr-3*, *mcr-8*, *mcr-10*) has also been developed (Hu et al., 2021b). A recent study has established a rapid, efficient and accurate method for recombinase polymerase amplification (RPA) combined with lateral flow dipstick (LFD) detection, but it can only detect the *mcr-1* gene. But *mcr-9* and *mcr-10* is also gradually being found in clinical patients and has spread widely around the world (Carroll et al., 2019; Ling et al., 2020; Liu et al., 2021). These methods can only detect some of the *mcr* genes, and some of them take a long time to detect. Therefore, it is essential to develop rapid detection methods that can cover all of the reported MCR family genes.

In this study, we selected the conserved region of all MCR family genes as the target sequence, considering all available relevant variant sequences in NCBI (as of March 2022), and successfully developed a multiplex fluorescent qPCR assay for the simultaneous detection of *mcr-1* to *mcr-10* gene sequences by optimizing the reaction amplification system. The results showed that the newly developed assay is highly sensitive, specific, and reproducible. Using a common 96-well instrument, 24 samples can be detected in a single experiment, and if a 384-well instrument is used for high-throughput detection, more strains can be detected simultaneously, which is beneficial for the processing of large number of samples.

To our knowledge, this is the first multiplex TaqMan fluorescence qPCR assay for all 10 MCR family genes. The assay provides a rapid, simple, sensitive, and specific technique for monitoring multidrug-resistant bacteria carrying the *mcr* gene. During the course of the study, six newly identified *mcr* gene variants (*mcr-1.35*, *mcr-1.36*, *mcr-3.42*, *mcr-4.7*, *mcr-4.8*, *mcr-5.5*) were discovered between April 2022 and November 2023. We found that the mutant base of the new *mcr* variants did not appear at the location where we designed the primer, so these new *mcr* gene variants could still be specifically amplified with the existing primer and probe sets. This indicates that the target sequence is relatively conservative in these variants, and also validates the reliability of the sequence chosen for our design. We believe that the new approach may apply to the *mcr* gene variants that emerge in the future.

## Data availability statement

The original contributions presented in the study are included in the article/Supplementary material, further inquiries can be directed to the corresponding authors.

## Author contributions

XG: Methodology, Writing – original draft, Data curation, Investigation. GY: Data curation, Methodology, Resources, Supervision, Writing – review & editing. WL: Data curation, Methodology, Resources, Supervision, Writing – review & editing. DW: Data curation, Investigation, Methodology, Writing – review & editing. CD: Data curation, Investigation, Methodology, Writing – review & editing. XJ: Data curation, Investigation, Methodology, Writing – review & editing. ZL: Data curation, Investigation, Writing – review & editing. XZ: Investigation, Validation, Writing – review & editing. RY: Investigation, Validation, Writing – review & editing. DZ: Methodology, Project administration, Resources, Supervision, Writing – review & editing. YW: Methodology, Project administration, Resources, Supervision, Writing – review & editing.
