# Supplementary material for: A multiplex TaqMan real-time PCR assays for the rapid detection of mobile colistin resistance (mcr-1 to mcr-10) genes
Source: Front Microbiol. 2024 Mar 13;15:1279186. doi: 10.3389/fmicb.2024.1279186 (PMC10967403; doi:10.3389/fmicb.2024.1279186)
Supplement: Supplementary file 1 [file Table_1.DOCX]

Supplementary Material

##
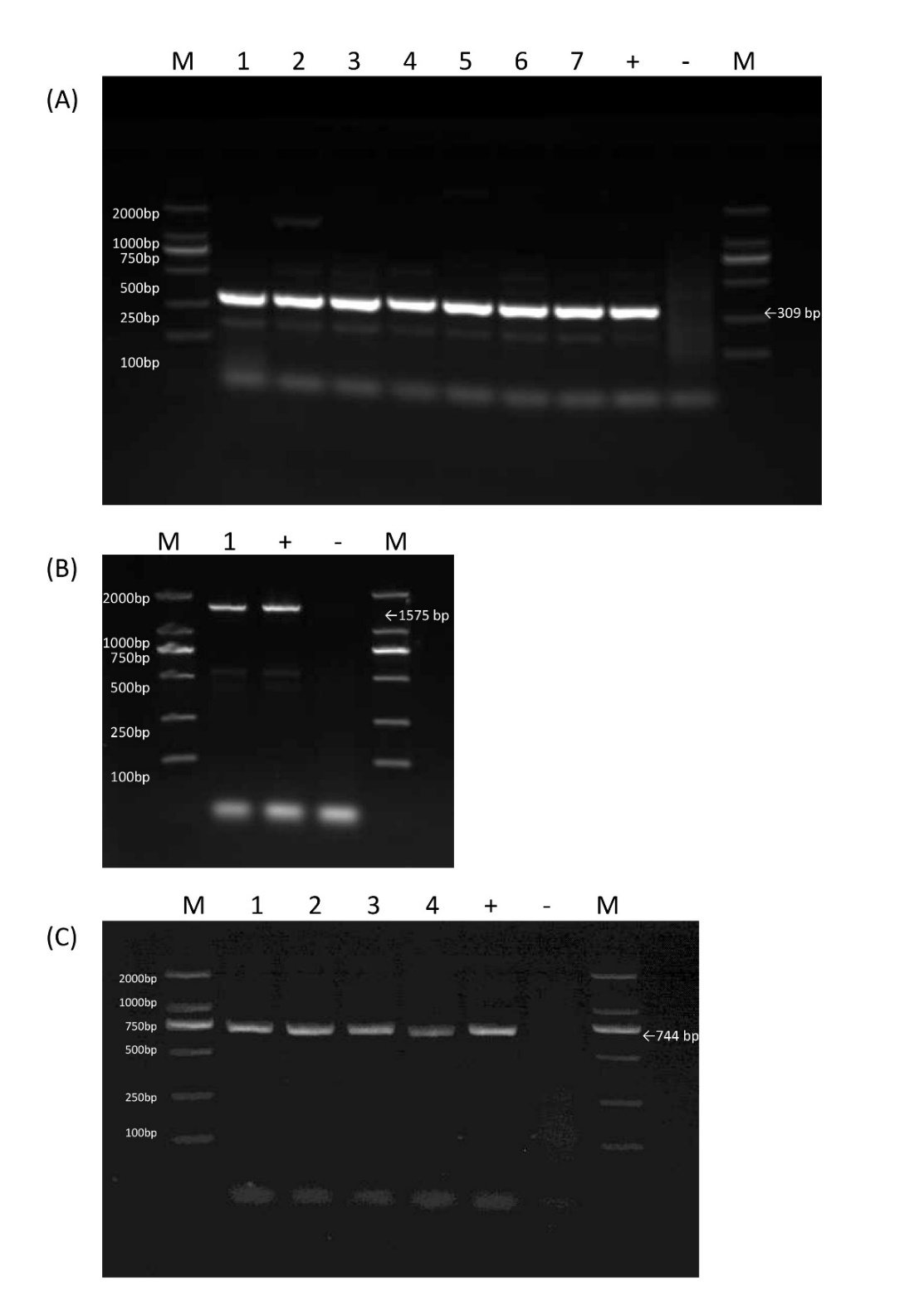
Supplementary FiguresSupplementary Figures 1：

(A) electrophoresis results of *mcr-1* positive strains (B) electrophoresis results of *mcr-9* positive strains (C) electrophoresis results of *mcr-10* positive strains
